# First-line managers’ experiences of and reflections on structural conditions for management practice in hospital settings

**DOI:** 10.1108/LHS-07-2024-0060

**Published:** 2024-12-20

**Authors:** Karin Lundin, Bernice Skytt, Marit Silén, Maria Engström, Annika Strömberg

**Affiliations:** Department of Health and Caring Sciences, Faculty of Health and Occupational Studies, University of Gävle, Gävle, Sweden; Department of Caring Sciences, Faculty of Health and Occupational Studies, University of Gävle, Gävle, Sweden and Department of Public Health and Caring Sciences, Faculty of Medicine, Uppsala University, Uppsala, Sweden; Department of Health and Caring Sciences, Faculty of Health and Occupational Studies, University of Gävle, Gävle, Sweden; Department of Health and Caring Sciences, Faculty of Health and Occupational Studies, University of Gävle, Gävle, Sweden and Nursing Department, Medicine and Health College, Lishui University, Lishui, China; Faculty of Education and Business Studies, University of Gävle, Gavle, Sweden

**Keywords:** Hospitals, Leaders, Management, Working conditions, Qualitative research, Nurses

## Abstract

**Purpose:**

The purpose of this paper is to describe first-line managers’ (FLMs’) experiences and reflections on structural conditions for management practice within hospital settings using Kanter’s theory of structural empowerment.

**Design/methodology/approach:**

A qualitative deductive approach with a descriptive design was used. Interviews were conducted with 11 FLMs in charge of medical or surgical hospital units spread across Sweden. Data were analyzed using a directed content analysis, based on Kanter’s theory of structural empowerment, encompassing such as access to necessary and sufficient resources, information, support and opportunities to learn and develop.

**Findings:**

Findings of this study from the FLMs’ descriptions and reflections shed light on the impact of power dynamics on the structural conditions for management practice. The availability of nursing staff was a fundamental resource in the FLMs’ work performance, ensuring delivery of care to patients and a sound work environment for staff. Additionally, the other structural elements outlined in Kanter’s theory were evident in the findings, as the FLMs wished for structured information flow, identified potential and challenged opportunities for development and emphasized the importance of receiving support from people with a genuine understanding of their work situation.

**Originality/value:**

The results of this study contribute to the understanding of FLMs’ structural conditions for management practice in hospital settings. The paper’s originality stems from the use of a deductive approach, providing a structured lens with the potential to inform future research and practice in the field of health-care management.

## Introduction

**Figure 1.**
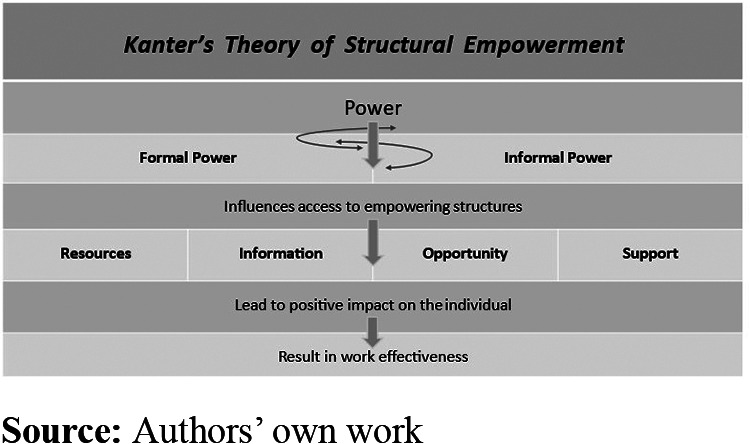
Kanter’s theory of structural empowerment

The work environment within the hospital context is challenging and multifaceted (De Vries *et al.*, 2023), and the work of first-line managers (FLMs) responsible for nursing staff and patient care has been described as complex (Figueroa *et al.*, 2019; Gunawan and Aungsuroch, 2017). FLMs have an important role, as they can influence the working conditions for nursing staff (Bawafaa *et al.*, 2015; Cummings *et al.*, 2018) and, thereby, the quality of the care delivered to patients (Sfantou *et al.*, 2017). Their position, between frontline staff and upper management, is key to how the organization’s goals and changes are communicated and implemented at the unit level (Ellis *et al.*, 2023). Reports of FLMs experiencing heavy workloads, limited authority in decision-making processes (Labrague *et al.*, 2018), high levels of emotional exhaustion and risk of burnout (Remegio *et al*., 2021) highlight the need for a better understanding of FLMs’ structural conditions for management practice. The role of FLMs in managing nursing staff includes formal duties such as staffing, budgeting, coordinating care and ensuring compliance with policies (Ericsson and Augustinsson, 2015; Gunawan and Aungsuroch, 2017). Within the FLM role, leadership is integral, as the FLMs guide and support staff to achieve care goals (Mintzberg, 1994). This study focuses on the structural conditions for FLMs’ management practice, where leadership is seen as an integrated component rather than an isolated phenomenon (Yukl, 2013). Structural conditions for management practice are, in this study, defined based on the empowering structures described in Kanter’s theory of structural empowerment (Kanter, 1993; Laschinger *et al.*, 2010). Kanter’s theory describe contextual factors within an organization that can promote a healthy work environment for the individual, influencing commitment and organizational efficiency (Figure 1). The theory suggests that an individual’s behavior and attitude toward work are shaped by access to empowering structures, rather than personality or abilities. These structures include access to *resources* such as materials, supplies, personnel and time, relevant and updated *information*, *opportunitie*s for learning and career development and *support* from superiors, colleagues and subordinates. Individuals with access to these structures are considered empowered. Access to these structures is influenced by *formal power*, derived from visible work roles that includes mandates, and *informal power*, derived from network of alliances within and outside the workplace. Kanter (1993) describes *power* as the ability to get things done, mobilize resources and obtain whatever is necessary to achieve one’s goals. Further, Kanter describes how power could be seen as existing in cycles, where access to power likely brings more power in ascending cycles and vice versa.

Kanter’s theory has been supported in studies within the health-care sector (Hagerman *et al.*, 2017; Haugh and Laschinger, 1996), linking FLMs’ perceived structural empowerment to their staff’s perceptions of structural empowerment. Several studies support links between perceived access to empowering structures and positive hospital nursing staff outcomes: lowered risk for burnout (Orgambídez and Almeida, 2019), perceived patient-centeredness of care (Goedhart *et al.*, 2017), increased work motivation and reduced occupational stress (Saleh *et al.*, 2022). Further, structural empowerment has been identified as a determinant of FLMs’ job satisfaction (Penconek *et al.*, 2021). However, qualitative studies that have used Kanter’s theory within the hospital context are rare. There are descriptions of how internationally educated hospital nurses perceive access to structural empowerment (Eriksson and Engström, 2018) and how FLMs in hospitals promote their nursing staff’s access to empowering structures (Lundin *et al.*, 2022). Less light has been shed through the lens of Kanter’s theory on the perceived access to empowering structures that hospital FLMs themselves experience in managing units and leading nursing staff.

Empirical evidence and theoretical reasoning underscore the significant role FLMs play in shaping the working life of nursing staff and in achieving organizational goals, including efficiency, job satisfaction among nursing staff and quality of care. As structural empowerment has been identified as a key determinant of FLMs’ job satisfaction, understanding FLMs’ experiences and reflections on access to empowering structures in their management practice is essential, for not only their work-life experience but also their staff and the organization. Furthermore, there is a lack of descriptive studies that provide a deeper understanding of these dynamics. Examining FLMs’ own experiences regarding their access to empowering structures can capture detailed narratives that enhance the current literature and provide valuable perspectives to inform both practice and future research. Moreover, the constantly evolving nature of hospital care and health-care organizations necessitates ongoing examination of structural conditions across various settings and perspectives. To contribute to the body of knowledge, a deductive approach is well-suited to explore whether existing theories or models hold true in different contexts (Patton, 2002). Consequently, the aim of this study was to describe FLMs’ experiences and reflections on structural conditions for management practice within hospital settings using Kanter’s theory of structural empowerment.

## Methods

### Design

A descriptive design with a deductive qualitative approach was used.

### Sample and setting

Striving for a sample consisting of FLMs working at different hospitals spread across Sweden, the sample was derived from previously randomized medical and surgical units participating in a national study conducted by the research group (Lundin *et al*., 2024). For the present study, 33 heads of department were identified and asked to give permission to contact FLMs at the randomized units again for an interview (*n* = 38). In all, 24 agreed and 25 FLMs received study information. Of all, 14 agreed to participate and 11 were included, with inclusion ending when data saturation was achieved. To be included, over one year’s experience as a hospital FLM was required. The FLMs managed units within medical (*n* = 5) or surgical clinics (*n* = 6) at university hospitals (*n* = 3), county hospitals (*n* = 6) or district hospitals (*n* = 2), spread across Sweden. The number of employees ranged between 21 and 100. A variation of practicing shared leadership (*n* = 3) and having an assistant manager (*n* = 5) existed among the participants. Their experience as FLMs at their current units ranged from 1 to 4 years (mean = 2 years) with four having prior FLM experience. Most participants were female (*n* = 10), and nine were educated nurses.

### Data collection and analysis

Data were collected through semi-structured interviews during late December 2023–April 2024. The interviews were performed by the first author (RN, lecturer and PhD student), who had previous experience of performing qualitative research and working within hospital settings. The interviews took place on a digital videoconference platform, lasted between 47 and 77 min (mean = 62 min) and were audio-recorded. A semi-structured interview guide with open-ended questions allowed the participants to speak freely about their experiences and reflect upon their structural conditions for management practice. Probes encouraged the participants to elaborate toward an enhanced depth and detail of their descriptions (Patton, 2002) (Examples of interview questions and probes):

1. Examples of interview questions:
  - What is your experience and view on the existing structural conditions for performing your work?
  - For FLMs in units like yours to effectively perform management and leadership, what structural conditions should be in place, in your experience?
2. Examples of probes:
  - What kind of support is it that you mean you are lacking?
  - What do you think has been important for you in achieving that at your unit?

Initially, participants described their background and entry into their current FLM role.

The recordings were transcribed using AI-powered software (Amberscript, Global B.V, 2024), checked for accuracy by the first author, who listened to the recordings several times and corrected errors. In parallel with the data collection, a directed content analysis (Assarroudi *et al.*, 2018) based on Kanter’s theory (Kanter, 1993; Laschinger *et al.*, 2010) was performed using NVivo 14 software (QSR International Pty Ltd, 2023). The first author identified meaning units in the transcripts that related to the research aim and assigned them to a categorization matrix derived from concepts within Kanter’s theory of structural empowerment, as described by Kanter (1993) and Laschinger *et al.* (2010). The categorization matrix included six categories: *resources*, *information*, *opportunity*, *support*, *informal power* and *formal power*. Some meaning units were more difficult to assign to a category within the categorization matrix as they encompassed descriptions where two or more empowering structures interacted with each other. Such units were at first sorted into a category named *unclear.* Then, each category was organized, with codes allocated to the meaning units that provided more detailed insights, enabling identification of patterns of similarities and differences. As an example from the analysis, the meaning unit “You get a different understanding for the answer when you hear it from the person who made the decision. But we never did that, we only got someone who was mediating or conveying the decision. Why couldn’t we cut back on weekly hours and use them on weekends more? And why didn’t we get compensation for the weeks moved outside the vacation period? We just got ‘no, we won’t be getting into that now.’” (FLM07) was sorted into the category *information* and in the organization phase assigned the code *Limitations in information flow when shut out of decision-making process*. Meaning units that contained a mix of several empowering structures were, after the organization phase, sorted into the category that dominated the respective meaning unit’s content. An overarching description of what was seen to be present in and intertwined between the categories was formulated. The categorization was discussed with all co-authors until consensus was reached. When the researchers saw that the predetermined categories were sufficiently represented with a variation of differences and similarities, they determined that data saturation had been achieved (Saunders *et al.*, 2018). When subsequent interviews did not yield any additional insights or variations, inclusion of participants stopped and no further attempts were made to contact the three FLMs who initially accepted but did not schedule an interview.

### Ethical considerations

The Swedish Ethical Review Authority (Dnr 2023-05951-01) approved the study. All FLMs were given verbal and written study information and informed that participation was entirely voluntary and that they could withdraw from the study at any time without providing a reason. Further, they were assured of confidentiality. All FLMs provided written study consent.

## Results

FLMs’ descriptions of experiences and reflections on structural conditions for management practice are presented in six categories. The boundaries between the categories are not always distinct, as interactions between the described structural conditions occurred in the data. The six structures from Kanter’s theory (Kanter, 1993; Laschinger *et al.*, 2010): resources, information, opportunity, support, formal power and informal power are represented in one category each. Before outlining the description of each category, an overarching description of what was seen to be present and intertwined in the categories is introduced.

### Power dynamics shape the structural conditions for management practice

The concept of power was found to permeate the FLMs’ descriptions of their structural conditions for management practice. Power was illustrated as being received through an interplay between formal and informal power. In their reflections, the FLMs described how they perceived access and lack of access to power. This influenced how the FLMs described their structural conditions. In their descriptions and reflections, their perceived access to power was sometimes related to the organization and sometimes specifically to their superiors.

#### Staff – the main resource for effective management practice.

The FLMs described the availability of staff as a central structural condition for their management practice. They reflected on how the availability of staff affected their work and their responsibilities for the work environment and patient care and it became apparent that they lacked control over the allocation and use of staff resources. They described navigating within a large organizational framework where strategic decisions were made at higher levels, excluding their direct involvement. These strategic decisions concerning allocation and use of staff resources subsequently impacted their units. One example described was when the Swedish health-care region’s strategy of managing the shortage of nurses led to an embargo on temporarily hiring nurses from staffing agencies:

As far as I know, there are no strategies for the summer. Even though we have used temp staff during the summer. We haven’t gotten any other tools. How do we solve it without temp staff? It’s largely up to us to solve operations with the resources we have. (FLM07)

In their reflections on the strained situation resulting from staff shortages, they described how units with stable staffing were affected by the organization’s general staff shortage and the resulting bed shortage. They emphasized the need to guard against excessive adaptation to the prevailing situation, as this created a risk of losing focus on the unit’s main mission, thereby losing staff members who wanted to work specifically with the unit’s intended patient groups and care processes.

Conversely, the FLMs also provided positive examples where their participation in strategic planning concerning their unit’s staffing had led to the successful attainment of organizational goals. They highlighted the need for strategies to improve the work environment and practices in the long term, implementing better systems to anticipate and plan for future staff and care needs:

When I came here, we were dependent on temp staff and I said to my closest superior: “I don’t know anyone here but give me two years.”//When I sent the recruitment request, to be allowed temp staff, my justification was pretty clear and I wrote that if I’m running an operation that never closes any beds, year round, then I need to plan ahead//me getting approval doesn’t mean I’ll automatically get all that temp staff, but it gives me the possibility to plan ahead//I think that made the highest manager believe my justification and she helped me and at the same time I showed that it did really take two years (to become independent of temp staff). (FLM11)

There were examples of how FLMs with a nursing background viewed themselves and were viewed by others as a resource that could be used to cover for insufficient staffing in urgent situations and during the summer period. It was described by some FLMs as their own choice and by others as something required by top management during strained times.

Other resources linked to managing staff-related responsibilities, such as having someone to share responsibilities with or delegate certain tasks to was described as especially important when having a large staff group. The FLMs underlined that without such functional support, managing large operations would be nearly impossible. They also described a need for more tailored support functions, pointing out that some existing systems were ineffective, like the example given from a region where a deputy only could act in an FLM’s absence, although the FLM advocated for closer collaboration and more consistent support to ease transitions and improve management efficiency.

Economic resource allocation was presented as another challenging structural condition for FLMs. They described difficulties balancing investments in personnel and equipment with limited and rapidly changing financial resources. They provided examples of seeking external funding for projects to improve the work environment and collaborating with other units to share costs, emphasizing a need for financial creativity to meet organizational goals.

#### Advocating for structure and flow – promoting timely and relevant information.

The FLMs described and argued for access to timely and relevant information to manage and lead their units. Large amounts of information flowed through various channels within the organization. This meant that a clearer structure was needed to facilitate handling and prioritization of what information should be communicated by FLMs to their staff when and how and also when and how they could expect to receive what information themselves. They believed that having a well-defined strategy for managing information would improve their efficiency and decision-making:

It’s also one of those things – what information can I share and when can I do that and when should I not say anything at all? (FLM01)

Further, they described how upper management’s withholding of information sometimes led to frustration and confusion and how structures set into place by superiors could prevent access to timely information:

But our superior is very conscientious about making that clear and has an established meeting structure, that we have certain meetings for certain things. And being very firm about that. Talking about operative matters at a strategy meeting: “No, we’ll deal with that on Tuesday.” Even if we’re squirming in our seats because there’s a fire here and now, we need to talk about it. No. We don’t do that. (FLM10)

They described an overflow of information by e-mail, meaning that important information might not be noticed in time. A hope that urgent information would be communicated verbally was expressed.

#### Imbalance between ambition, authority and practical challenges – impacting the opportunities for development.

The FLMs described various experiences of opportunities to develop and learn. They expressed a passion and desire to develop, focusing on how they thrived trying out new roles or structures within their organizations and creating opportunities for development and growth for their employees and unit. The opportunity to perform and succeed with such developmental work motivated the FLMs to stay in their role:

This is the kind of thing you feel like you’ve wanted, to retain or build or just to get the personnel team to function. It’s been super satisfying, what we’ve managed to do when it comes to the psychosocial workplace, I think. That’s probably why I’m still here. (FLM09)

They emphasized the importance of participating in training and leadership programs to learn the ropes of being a manager. They gave examples of such programs within the organizations but were sometimes prevented from participating because of daily responsibilities or superiors being opposed to individual FLMs request to participate, not acknowledging the FLM’s expressed need. FLMs who had had the opportunity to participate recognized the potential of the tools and knowledge acquired from these programs, but struggled to find time and resources to apply them in practice. Some described this as leading to shortcuts where changes were made without using established tools:

And then when you go to these training sessions, you think, like “We could easily make some changes or improvements in this area and that area. Then I get to use my tools and practice a little, while we also do something that is good for the unit.” But that’s over when you come back to reality. You realize that “Okay, when am I supposed to have time for this?”//If we’re going to succeed with any change or improvement, I don’t have time to focus on the tools – we’ll make a change and hope that we get an improvement. It’s a shame. (FLM07)

#### Key to successful support – a genuine understanding of the first-line managers’ complex scope of responsibility.

The support described by the FLMs as valuable and significant came from those who possessed and demonstrated a genuine understanding of the challenges that FLMs face in their day-to-day work. Emphasis was placed on the support received from colleagues in managerial roles, with an understanding and preferably with experience specific to the FLM level within the organization. Regular forums involving colleagues and *ad hoc* meetings with superiors were given as examples of structures that promoted FLMs’ opportunities for receiving support. Feedback and recognition from superiors and colleagues helped them feel appreciated and motivated in their roles and feedback from staff was described as valuable, as it helped them understand how they performed. Mentorship was described as another significant form of support. The availability of mentors varied between the organizations, some having established structures for mentorship, whereas others lacked such structures. This had led a few FLMs to arrange for unofficial mentors on their own. In some cases, a mentor compensated for the lack of support received from an FLM’s superior:

I can say that I’ve gotten a mentor, just because I want someone who knows stuff, she’s been a manager for a long time and is in the same area//Everything I’ve done, I’ve gotten confirmation from her. (FLM02)

FLMs without a nursing background and who were new to the hospital organization described having high perceived access to support and stated that this was seldom questioned by superiors, staff or others in the organization:

And since I’m completely green [in this kind of operation, in healthcare], no one thought it was weird that I had a bunch of questions, so it was… I could be entirely open about “I don’t know anything about this, can you help me?” and that was never an issue. Everyone was really helpful, so I’ve never felt abandoned or confused, there’s always been someone who could step in and help. (FLM11)

#### Awareness of limited formal power-maintaining trust through clarity and acting within boundaries.

In their descriptions of structural conditions for management practice, the FLMs emphasized the importance of being acknowledged and seen as trustworthy and competent by the organization, their superiors and their staff. They described experiences of constraints and structured pathways inherent within the organizations. Their access to formal power in some cases operated within a rigid hierarchical framework, requiring them to follow a specific chain of command, involving multiple layers of approval:

We have a very siloed organization, so I need to go to my superior. He’s the assistant operative manager, but also head of the care departments. So he goes to the operative manager, who goes to the area manager, who might go to the director of healthcare. Then there’s a bunch of HR people up there too, who will look at it [the proposal]. So, I can’t contact the area manager, because that’s not on my line. I can talk to the operative manager, that’s not a problem, but there’s a lot of steps that need to be skipped there. (FLM03)

They described that being a manager at a unit acknowledged as contributing with a specialized and important function strengthened their mandate to influence their structural conditions in day-to-day operations:

I do think people listen to me. People show an understanding of the fact that we need the staffing that we have, and we’ve always been allowed to use temp staff when we feel it’s necessary. We’re kind of in between in-hospital care and an outpatient center. Because we do life support care, we’re kind of prioritized in a lot of ways, compared with other units. But that’s for a good reason, because we really need it, otherwise people will die. (FLM01)

The FLMs stated that acknowledgment from their superiors was important for them to be seen as belonging within the management group and for staff to perceive them as leaders.

Further, the FLMs described how high turnover among FLMs could result in staff not seeing them as trustworthy. The organizations’ different choices regarding employment forms for FLMs – short-term or permanent positions – were also reflected on. The short-term positions were seen as influencing the retention of FLMs in a negative way, offering an easy way out for an FLM when confronted with a sense of overwhelming challenges and for the employer in times of reorganization.

#### Alliances and networks – promoting influence and action through informal power.

The FLMs described how they actively worked on building relationships by asking questions, greeting people and being positively inclined toward cooperation. With this approach, they hoped to set a good example for others. They emphasized how personal contact could facilitate cooperation and communication. They stated that having established personal contact with someone from the finance or human resource department made it easier to ask for help compared with when interacting with someone they had never met. Having worked within the same organization before entering the role as an FLM made it easier to establish important contacts. FLMs without previous experience from their current organization emphasized the importance of networks to get their job done:

So a lot is about building a contact network and building trust for the people I need to collaborate with in order for things to work. (FLM04)

Additionally, they described how their access to alliances with other specialties was important for direct patient care, facilitating their staff’s consultation with other units when needed. Creating forums for such alliances to be established was seen as something the FLMs could be responsible for:

It’s more important that it works for the staff who actually work closely with patients; that they can call and consult if needed, or if they have a patient who has attempted suicide, they can get support from psychiatry on how to handle things. In that case, of course, I can create a forum for that. (FLM06)

## Discussion

The structural conditions described by the FLMs aligned with the empowering structures in Kanter’s theory (Kanter, 1993; Laschinger *et al.*, 2010). Also, in line with Kanter’s theory, access to power emerged as a more overarching description, with the dynamic between access to formal and informal power permeating the participants’ descriptions of access to the other four structures: resources, information, opportunity and support. The primary focus in the FLMs’ reflections was on how their structural conditions influenced their work with creating a sustainable work environment for their staff and ensuring safe and high-quality care for patients.

### Contribution and alignment to research and theory

The findings correspond with previous research with a deductive approach showing the importance of access to structural empowerment within health-care settings from the perspectives of FLMs in elderly care (Skytt *et al*., 2015) and hospital nursing staff (Eriksson and Engström, 2018; Lundin *et al.*, 2022). The FLMs’ articulated their experiences and reflections on structural conditions, providing specific examples of situations and work tasks that reflect their complex and challenging responsibilities, as noted in other studies (Ericsson and Augustinsson, 2015; Gunawan and Aungsuroch, 2017). When they felt successful in managing their units, they described a positive impact on their own work situation (Keith *et al.*, 2021). Their descriptions and reflections align with how Kanter’s theory describes structural empowerment and the concept of power in ascending and descending cycles (Kanter, 1993). FLMs’ access to resources dominated the descriptions in our findings, and as resources often can be and are limited, hospital organizations must work with what is available (Drennan and Ross, 2019). However, our findings revealed how that work could be more or less effective depending on if FLMs were given the opportunity to influence their own scope of action, with the mandate to act more independently and permission to participate in decision-making processes (Trus *et al.*, 2012). The findings provided good examples of when FLMs were allowed to influence decision-making processes, resulting in achievement of organizational goals. Those examples could illustrate structural empowerment in action or be labeled as examples of shared or professional governance (Clavelle *et al.*, 2016). Magnet hospitals are organizations practicing such non-hierarchical governance and are associated with higher ratings of the nursing staff’s work environment and quality of care than non-Magnet hospitals (Rodríguez *et al.*, 2020). The professional practice environment at Magnet hospitals has been shown to provide their employees with a voice in practice policies and organizational decisions, promoting autonomy and giving recognition for their contributions aligning with the empowering structures in Kanter’s theory (Laschinger *et al.*, 2003). Further, our findings could be discussed in relation to the concept of psychological empowerment, being that the FLMs’ experiences of having opportunities to act independently, participate in decision-making processes and receiving support for their ideas, may reflect key elements of psychological empowerment, encompassing meaning, competence, self-determination and impact (Spreitzer, 1995). The positive relationships between levels of structural and psychological empowerment within nursing is studied from the perspective of both FLMs and nursing staff (Wagner *et al.*, 2010) and nurses alone (Fragkos *et al.*, 2020). Laschinger’s and colleagues’ integrated model of nurse/patient empowerment further argue that nursing work environments offering access to empowering structures contribute to higher psychological empowerment of nurses, leading to higher patient empowerment, resulting in better patient outcomes (Laschinger *et al.*, 2010). Quantitative studies support this integrated model, for example by showing significant relationships between nursing staffs’ access to empowering structures and hospital patients satisfaction (Donahue *et al.*, 2008) and ratings of older persons satisfaction with care (Engström *et al.*, 2021). As Kanter’s theory (Kanter, 1993) and empirical findings (Hagerman *et al.*, 2017) point to relationships between FLMs’ perceived access to empowering structures and that of their staff, organizations would benefit from establishing and maintaining accessible empowering structures for FLMs.

### Making power dynamics visible to promote access to empowering structures

Power could be seen as a catalyst for getting the job done (Kanter, 1993) with the best possible outcomes for all health-care personnel and patients (Laschinger *et al.*, 2010). Our findings included examples where a lack of power inhibited FLMs from accessing the organizations’ established structures for development and support. In some descriptions, the closest superior emerged as a gatekeeper of both formal and informal power. FLMs’ superiors should be aware that they are in a position to influence FLMs’ access to empowering structures (Hagerman *et al.*, 2017; Haugh and Laschinger, 1996). Promoting FLMs’ access to formal and informal power is something that both superiors and the organizations can work on (Kanter, 1993). Another aspect related to FLMs’ perceived power in our findings was employment form. At Swedish hospitals, FLMs often are employed as nurses, with a managerial assignment typically lasting between three and five years. This system was seen as designed for a high turnover of managers when reflected upon by some of the participant FLMs. Further, our findings of how the FLMs described a variety of individual needs, for example, access to support and opportunities to develop and learn could question hospital organizations having a “one size fits all” design for such structures (Liang *et al.*, 2020; Sisk *et al.*, 2021). In the present study, the participating FLMs without a nursing background could be seen as tokens, a perspective of Kanter’s theory referring to individuals from minority groups being given positions of authority within an organization yet facing challenges in exerting any true influence (Kanter, 1993). Kanter describes how tokens may feel isolated or marginalized, lacking the support or resources to effect meaningful change. In contrast to Kanter’s assumptions, the FLMs without a nursing background did not describe having less power than other FLMs. They perceived themselves as acknowledged and listened to, despite lacking prior nursing or hospital experience, and described their support needs being met, without any disadvantages related to being tokens. Our participants emphasized that while nursing expertise was important, understanding the bigger picture and drive unit-wide improvements for staff and patients was even more vital. Existing studies exploring the token perspective within health-care settings are sparse (Finegan and Spence Laschinger, 2001; Hagerman *et al.*, 2015). However, there are some findings in previous research (Hagerman *et al.*, 2015) that could – along with the descriptions from our participants – call into question Kanter's (1993) negative portrayal of tokens. Hence, further exploration would be interesting, with future quantitative studies comparing perceived access to empowering structures among FLMs with and without a nursing background, and qualitative studies investigating diverse experiences and perspectives on power dynamics.

### A need for broader perspectives to understand contextual aspects influencing management practice

The complex organizational context in which hospital FLMs operate necessitates viewing their unit-level work in relation to the broader setting. The findings of this study highlighted the challenges FLMs face in achieving long-term, stable planning, for example, as regards staffing, when other units within the same organization were experiencing significant resource shortages. Organizations must be capable of shifting focus between overarching goals and the impact of these on individual units. Different management perspectives likely exist at different levels within an organization. Previous research indicate that FLMs sometimes struggle with differences between their own perceived management perspective, viewed as value-orientated, and the central management perspective, seen as more production-oriented (Strömberg *et al.*, 2019). Positioned at this intersection, FLMs could provide valuable insights in forums where the organizations’ resource allocation and strategic planning are discussed, fostering a balance and responsiveness to both macro-level objectives and micro-level operational realities within direct patient care. The fundamentals of care framework outlines these dual perspectives, focusing on succeeding in meeting patients caring needs (Feo *et al.*, 2018), which could be seen as hospital organizations’ overarching goal. Within that framework, the dimension of context encompasses four system-level enablers – resources, culture, leadership and evaluation and feedback – and four policy-level enablers: financial, quality and safety, governance and regulation and accreditation (Kitson *et al.*, 2013). These enablers appear to align with and reinforce the theoretical framework of structural empowerment used in the current study (Kanter, 1993; Laschinger *et al.*, 2010). Further investigation into this alignment could enhance understanding of how contextual aspects influence health-care personnel having the resources, support and environment needed to perform their duties effectively and confidently.

### Methodological considerations

A limitation of the study is its focus on FLMs in a specific health-care system, which may limit transferability. However, the similar responsibilities of hospital FLMs overseeing nursing staff worldwide suggest relevance in other contexts. Despite conducting fewer interviews than initially planned, because of limited access to additional participants, the study was strengthened by the diversity of the sample within the specific context, capturing a range of perspectives and experiences that enhance the transferability of the findings and reflect the complexities and variations in the phenomenon of hospital FLMs’ structural conditions. The findings showcase both differences and variations while also revealing recurring common reflections, thereby enhancing credibility. Another strength is the deductive approach, limiting subjective interpretation and increasing the reliability and relevance of the research for practice. The detailed description of the research process supports dependability, and representative quotations support credibility.

## Conclusions

Hospital FLMs emphasize the importance of having access to empowering structures, in line with Kanter’s theory. They highlight that these structural conditions are essential to fully meet the expectations placed on their management practice by their organizations, their staff, their superiors and themselves. Their experiences highlight how access to formal and informal power, along with resources, information, opportunities and support, strengthens their ability to effectively execute their roles and responsibilities. Their descriptions underline how power dynamics relate to the accessibility of empowering structures in practice.

## Implications for practice

By bridging the gap between theory and practice, this research contributes to the body of knowledge describing the importance of FLMs’ access to empowering structures. It can be used in teaching to inform future FLMs in health care and influence public policy by highlighting the need for structural empowerment in health-care settings. Specifically, our findings suggest that actions are needed at multiple levels: At the managerial level, superiors can facilitate FLMs’ navigation within power dynamics. At the organizational level, transparent strategies for facilitating FLMs’ participation in networking forums, promoting their informal power. To strengthen FLMs’ formal power, clarify and align the roles and responsibilities of FLMs and give FLMs a seat at the table in hospital organizations, acknowledging their operational expertise. Ensure employment forms for FLMs signal recognition and long-term investment promoting formal power while also having potential economic impacts by promoting retention. At the national level, create arenas and forums to discuss nationwide health-care challenges, fostering unified strategic plans that impact FLMs’ daily operations, nursing workforce and patient care.
